# Lightweight Federated Learning Approach for Resource-Constrained Internet of Things

**DOI:** 10.3390/s25185633

**Published:** 2025-09-10

**Authors:** M. Baqer

**Affiliations:** Department of Computer Engineering, College of Information Technology, University of Bahrain, Sakhair P.O. Box 32038, Bahrain; mbaqer@uob.edu.bh

**Keywords:** federated learning, internet of things, wireless sensor networks, artificial intelligence, machine learning, energy-efficient learning, nearest neighbor, pattern recognition, in-network processing, communication efficiency

## Abstract

Federated learning is increasingly recognized as a viable solution for deploying distributed intelligence across resource-constrained platforms, including smartphones, wireless sensor networks, and smart home devices within the broader Internet of Things ecosystem. However, traditional federated learning approaches face serious challenges in resource-constrained settings due to high processing demands, substantial memory requirements, and high communication overhead, rendering them impractical for battery-powered IoT environments. These factors increase battery consumption and, consequently, decrease the operational longevity of the network. This study proposes a streamlined, single-shot federated learning approach that minimizes communication overhead, enhances energy efficiency, and thereby extends network lifetime. The proposed approach leverages the *k*-nearest neighbors (*k*-NN) algorithm for edge-level pattern recognition and utilizes majority voting at the server/base station to reach global pattern recognition consensus, thereby eliminating the need for data transmissions across multiple communication rounds to achieve classification accuracy. The results indicate that the proposed approach maintains competitive classification accuracy performance while significantly reducing the required number of communication rounds.

## 1. Introduction

Over the past few years, federated learning has become a widely adopted paradigm for distributed, collaborative machine learning, particularly suitable for privacy-sensitive applications [1,2,3,4,5]. Originally introduced by Google, federated learning shifts the focus from centralized data processing to on-device computation, enabling devices to exchange only model updates rather than raw sensor data [6]. In federated learning, devices collaboratively construct a global model by communicating locally computed model updates instead of exchanging raw data, thereby preserving user privacy. This process iteratively improves the global model while keeping raw data local, reducing the need to transmit large amounts of sensor data across the network to the server or base station [1,2,7,8]. The key characteristics of federated learning include scalability, device and statistical heterogeneity, and data privacy [1,9].

The performance of federated learning is often comparable to—and in some cases surpasses—that of traditional centralized machine learning approaches [7]. Comparable principles have also been observed in graph neural network research [10,11,12,13], where distributed learning is employed to optimize communication efficiency and maintain data locality. Traditional machine learning approaches require substantial computational power, memory storage, and large training datasets [2,14]. Moreover, conventional CPUs or GPUs are generally unavailable in IoT devices and are unsuitable for untethered and battery-powered deployments [2]. Crucially, most machine learning methods rely on centralized processing, necessitating that both data and models reside in a single location [1,14]. Federated learning algorithms enable multiple nodes to collaboratively train a global model in a fully distributed manner without communicating raw data to a central location for processing [2,15].

Federated learning has been proposed as an effective solution for distributed information processing in the Internet of Things (IoT). The IoT represents a transformative, ubiquitous paradigm that has been seamlessly integrated into the fabric of everyday life [16,17,18,19,20]. Initially, the term IoT was associated with radio frequency identification (RFID) tags [21]. However, the current IoT paradigm encompasses a diverse range of interconnected devices, including near-field communication (NFC) devices, sensors, actuators, wireless sensor networks (WSNs), and smartphones. IoT devices are now pervasive in daily life and influence decision-making, behavior, and lifestyle [21]. The application domains of the IoT are vast and continually expanding across various domains and sectors [21,22].

This study focuses on WSNs and wireless IoT (WIoT) devices, both of which form important subsets of the broader IoT domain. These devices act as bridges between the physical and digital environments by acquiring sensor data and transmitting it to other IoT nodes or end-users [16,17,18,23,24,25,26,27,28,29,30,31,32]. Typically constrained by limited processing power, memory capacity, and battery life [21], WSNs and WIoT devices are often deployed for remote sensing in locations where battery replacement or physical maintenance is impractical or infeasible. In such scenarios, energy efficiency becomes a critical design objective, directly affecting network lifespan and operational reliability [33,34].

To ensure reliable operation, IoT applications must optimize processing power, memory storage, and communication overhead to conserve the limited battery energy of the IoT devices [16]. Sensor data collected from IoT environments require local storage, processing, and communication; however, these processes are constrained by limited device resources [24]. Furthermore, IoT network communication is often highly dynamic, less reliable, and slower than that of traditional infrastructure, making the assumption of continuous node availability unrealistic in many deployment scenarios [1]. Beyond device-level challenges, large-scale IoT deployments exert heavy demands on communication networks and end-systems such as base stations and servers.

Technological advances have driven the rise of federated learning-enabled IoT edge computing, offering efficient solutions and innovative applications [35]. This synergy enables diverse applications: in healthcare, federated learning–IoT integration accelerates patient diagnosis and treatment while preserving data privacy [36]. Specifically for medical IoT, federated learning enhances both privacy and classification accuracy in applications such as chronic disease prediction [37,38]. Furthermore, federated learning is increasingly applied in smart city domains such as traffic management and smart vehicles, where federated learning enables collaborative solutions including an improved recognition of unlabeled traffic signs among vehicles and a more efficient identification of charging stations [39,40].

Recent research has explored lightweight and secure distributed learning paradigms that align operational constraints with the approach proposed in this study. For example, the combinatorial optimization graph neural network (CO-GNN) employs graph neural networks (GNNs) to jointly optimize beamforming, power allocation, and intelligent reflecting surface (IRS) phase shifts in non-orthogonal multiple access (NOMA) systems, achieving secure, resource-efficient transmission without explicit channel state information (CSI) estimation [41]. Similarly, the work in [42] introduced a safety-optimal, fault-tolerant control method for unknown nonlinear systems with actuator faults and asymmetric input constraints, ensuring both safety and optimality. This approach integrates control barrier functions, neural network-based system identification, and an adaptive critic scheme to guarantee stability and efficiency. Furthermore, the authors in [43] proposed a collaboration-based node selection (CNS) approach that enhances wireless security by combining relay selection with friendly interference, thereby improving secrecy and reliability through selection combining (SC) and maximal ratio combining (MRC) strategies, with performance validated through both theoretical analysis and simulation.

Despite extensive research on federated learning and the IoT, achieving result model convergence and acceptable accuracy often requires a large number of communication rounds, during which local model updates from active network nodes are communicated throughout the network and aggregated at a central server or base stations. These iterative communication rounds introduce substantial communication overhead and impose significant energy demands on participating nodes. These limitations underscore the need for energy-efficient strategies to improve federated learning performance and the lifetime of the network. Consequently, the implications of communication overhead and energy consumption remain underexplored in the context of resource-constrained IoT environments. The main contributions of this paper are as follows:

- Novel Federated Learning Approach: A federated learning approach is proposed, namely the tiny federated nearest neighbors (TFNN) approach, tailored for IoT devices and networks that significantly reduces communication overhead, computational load, and energy conservation—critical considerations in resource-constrained environments.
- Single-shot Communication: This study leverages the *k*-nearest neighbors (*k*-NN) algorithm to perform collaborative pattern recognition in a single communication round, thereby minimizing communication overhead.
- Performance Benchmarking: TFNN is benchmarked with the classical *k*-NN and federated learning baseline algorithms, demonstrating that TFNN achieves competitive accuracy while requiring only a single communication round, in contrast to the multiple rounds typically needed by conventional federated learning approaches.

The rest of the paper is organized as follows: Section 2 provides an overview of related work on federated learning and *k*-NN. Section 3 describes the proposed TFNN approach in detail. Section 4 outlines the evaluation methodology, and Section 5 presents the simulation results and performance analysis. Finally, Section 6 concludes the paper by summarizing the key findings, discussing limitations, and proposing future research directions.

## 2. Related Work

This study explores the synergy between the *k*-NN algorithm and federated learning to enable distributed, collaborative pattern recognition in IoT systems. Before presenting the TFNN approach, we review the principles of *k*-NN and federated learning and examine how these algorithms interact in the context of distributed sensing.

### 2.1. k-Nearest Neighbors

The *k*-NN algorithm, originally introduced by Fix and Hodges [44,45], is a supervised non-parametric method widely employed in diverse classification problems. Its appeal stems from the absence of a dedicated training stage, its straightforward implementation, and its ability to handle multi-classes. Instead of iterative parameter updates, the *k*-NN algorithm essentially memorizes the training dataset and applies distance metrics to classify new instances.

The *k*-NN algorithm is described by the pseudocode in Algorithm 1. The *k*-NN algorithm employs a training set *D* to perform pattern recognition. Each instance in *D* consists of a pair ($x_{i}$, $y_{i}$) that describes a pattern and its label. For a given target sample *x* (i.e., the event pattern whose label is to be predicted), the algorithm computes the distance $d(x,x_{i})$ between *x* and each training pattern $x_{i}$ in D.

The distance metric can be defined using various methods, such as Euclidean distance, Manhattan distance, or other suitable distance measures. After computing the distance, the algorithm selects the *k* samples with the smallest distances. The parameter *k* specifies the number of nearest neighbors used to determine the label of the test sample.

Finally, the algorithm determines the majority label—the label that occurs most frequently—among the samples in *N* by counting the occurrence of each label *y* in $N_{k}$, as described in the following formula:

(1) $$\mathrm{Count}\left(y\right)=\sum_{j=1}^{k}I\left(y_{i_{j}}=y\right)$$

where I denotes the indicator function. The majority label is the label that appears most frequently among the *k^th^* nearest neighbors. The classified label ${\hat{y}}_{test}$ for $x_{test}$ is defined as follows:

(2) $${\hat{y}}_{test}=\arg\max_{y}\mathrm{Count}\left(y\right)$$

Formula (2) states that the predicted label for the test pattern is the label that occurs most frequently among its *k*-nearest neighbors. In other words, it selects the label with the highest count, i.e., the majority winning label. The *k*-NN algorithm is outlined in the pseudocode shown in Algorithm 1.
**Algorithm 1** k-NN algorithm.1:**Input**2:*D: a set of training samples* {*($x_{1}$, $y_{1}$),…,(($x_{n}$, $y_{n}$)*}3:*k: the number of nearest neighbors*4:**for** each training sample ($x_{i}$, $y_{i}$)∈ *D* **do**5:      compute $d(x,x_{i})$6:**end for**7:let *N* ⊆ *D* be the set of training samples with the *k* smallest distance8:**return** the majority label of the samples in *N*

### 2.2. Federated Learning

The federated averaging (FedAvg) algorithm [8] is a widely used aggregation algorithm in federated learning, where in each communication round, a subset of devices independently trains local models on private data. Instead of sharing raw data, these devices upload their local models to a central server, which produces a new global model by averaging the updates. This process iterates until the model converges to a satisfactory performance level.

A federated learning scenario for an IoT network consists of *N* nodes (IoT devices) and a centralized location for final processing—a server or base stations. Federated learning can be mathematically formulated as a distributed optimization problem for the global loss function $f_{\mathrm{FL}}$:

(3) $$f_{\mathrm{FL}}\left(\omega \right)=\frac{1}{K}\sum_{n=1}^{N}\sum_{k=1}^{K_{n}}f_{k}\left(\omega \right),$$

where ω represents the global model weights, *K* is the total number of data points across all devices, and $K_{n}$ denotes the number of data points stored on device *n*.

As described earlier, FedAvg is based on averaging the results of local stochastic gradient descent (SGD) updates, as summarized in Algorithm 2 [8]. The server selects a subset of nodes to participate in each round and distributes the current global model to all selected nodes. Upon receiving the global model, each node updates its own local model by performing the SGD procedure. The nodes then transmit their updated local models to the server, which computes a weighted average of the received updates to produce the new global model [46].

Initially, the global model $\omega_{0}$ is randomly initialized. In each round *t*, the server randomly selects a subset $S_{t}$ of clients (determined by a fixed participation fraction) and distributes the current global model $\omega_{t}$ to all nodes in $S_{t}$. All selected nodes operate in parallel, updating their local models according to $\omega_{t}^{k}\leftarrow \omega_{t}$. Each node then computes its local gradient descent step and sends its updated local model to the server. The server aggregates these updates to generate the new global model $\omega_{t+1}$.
**Algorithm 2** FedAvg algorithm.  1:**Aggregation Server**  2:Initialize $\omega_{0}$  3:**for** each round *t* = 1,2,… **do**  4:       Select a subset of clients $S_{t}$  5:       **for** each client *k* ∈$S_{t}$ **in parallel do**  6:             $\omega_{t+1}^{k}\leftarrow$ ClientUpdate(*k*, $\omega_{t}$)  7:       **end for**  8:       $\omega_{t+1}\leftarrow \sum_{k=1}^{K}\frac{n_{k}}{n}\omega_{t+1}^{k}$                  ▹ Averaging model updates from all selected clients  9:**end for**10:ClientUpdate(*k*, ω): // Run on client *k*11:**for** each local epoch *e* from 0 to *E* **do**12:      **for** each minibatch *b* of size *B* **do**13:            ω←ω−η∇ℓ(ω;b)                                                         ▹ Gradient descent update14:      **end for**15:**end for**16:return ω to the server

Nonetheless, FedAvg incurs substantial communication costs, since a large number of communication rounds are often required to achieve convergence. Moreover, local updates can sometimes cause FedAvg to diverge from the optimal global solution [47,48,49,50,51]. FedAvg also faces scalability challenges and may struggle to adapt to the dynamic conditions of IoT networks. In addition, its reliance on the arithmetic mean for model aggregation makes it vulnerable to data corruption and outliers [46,52].

FedProx, proposed in [53], extends the standard federated learning paradigm by introducing a proximal term into the local optimization objective function. This additional term discourages excessive deviation from the global model, thereby helping stabilize training in scenarios where participating IoT devices differ in computational capacity, data volume, or distribution. While this approach enhances robustness in non-IID conditions, it comes with notable trade-offs. The need to compute the proximal term increases computational workload on each device, and model behavior can vary significantly depending on the chosen regularization coefficient. Moreover, FedProx still requires multiple client–server communication rounds before convergence and remains dependent on the accuracy and stability of the global model used as a reference.

SCAFFOLD is a widely used federated learning algorithm designed to mitigate client drift caused by non-IID data [47]. It employs control variates—auxiliary vectors exchanged between the server and clients—to guide local updates toward the global optimum. SCAFFOLD offers provable convergence guarantees and performs effectively in heterogeneous data settings with significantly fewer communication rounds. Nonetheless, SCAFFOLD requires tens of communication rounds of model updates and the exchange of gradients and therefore still suffers from communication overhead and computational cost.

In general, it is important to note that achieving target accuracy in federated learning entails substantial communication overhead. Table 1 compares federated learning algorithms, namely FedAvg, FedProx, SCAFFOLD, and AdaptiveFL.

## 3. Tiny Federated Nearest Neighbors

This study proposes a federated learning nearest neighbor–based approach, referred to as TFNN, building upon the work presented in [55]. TFNN is designed to provide an energy-efficient federated learning solution for the IoT, explicitly addressing the memory, processing, and communication constraints of IoT nodes.

Pseudocode Algorithm 3 outlines the TFNN approach, in which IoT devices collaborate to transform their local inferences into a global event recognition outcome through decision fusion at the server or base station. In this approach, each IoT device stores training patterns locally and applies the *k*-NN algorithm to classify input test (event) patterns, as described in Algorithm 1.

In TFNN, the server first initializes the network by identifying the participating nodes, denoted as $N=\{n_{1},n_{2},\ldots ,n_{m}\}$. Each node independently executes a local inference process in parallel by invoking the Node_Update function, where *k* represents the number of nearest neighbors used in the local *k*-NN classifier. The parameter *P* is not transmitted; instead, each node is assumed to access the event pattern directly through local observation. The local prediction ${\hat{y}}_{i}$ from each node is then returned to the server.

Finally, the base station performs global decision fusion by applying majority voting over the class labels received from IoT nodes to produce the final predicted label $\left({\hat{y}}_{f}\right)$ for the event pattern $P_{t}$. Let $f_{i}\left(x\right)$ denote the class label of the ith nearest neighbors of *x*. The indicator function $\delta (c,f_{i}\left(x\right))$ outputs 1 if $f_{i}\left(x\right)=c$ and 0 otherwise.

(4) $$g\left(c\right)=\sum_{i}\delta \left(c,f_{i}\left(x\right)\right)$$

where g(c) denotes the number of neighbors labeled as class *c*.

(5) $$c^{*}=\arg\max_{c}g\left(c\right)$$

Here, $c^{*}$ represents the majority class label selected as the final prediction. The winning class is defined as the class receiving the majority of votes (50%+ϵ), as defined in Formulas (4) and (5).
**Algorithm 3** Tiny federated nearest neighbors (TFNN) approach.  1:Define set of active IoT nodes **Network Initialization:** $N\leftarrow \{n_{1},n_{2},\ldots ,n_{m}\}$  2:**Server/Base Station Executes**  3:**for** each node $n_{i}\in N$ in parallel **do**  4:      ${\hat{y}}_{i}\leftarrow \text{Node\_Update}\left(k\right)$                                                  ▹ Local classification via k-NN  5://communication step: each node sends only $\hat{y_{i}}$ (a class label) to server  6:**end for**  7:${\hat{y}}_{f}\leftarrow \mathrm{argmax}\sum_{i=1}^{m}\delta \left(c,{\hat{y}}_{i}\right)$           ▹ Server aggregates classifications via majority voting  8:function **Node_Update**(*k*)  9:${\hat{y}}_{j}\leftarrow \text{k-NN}\left(k,P_{local}\right)$                                            ▹ Perform location classification only10:return ${\hat{y}}_{j}$ to server

This federated learning design ensures that raw data is never exchanged, thereby preserving privacy and enhancing efficiency. Each node transmits only its winning class label, which the server aggregates through majority voting. This approach provides a lightweight, communication-efficient, and privacy-preserving federated learning mechanism well suited for low-power and resource-constrained IoT environments.

## 4. Evaluation

Assessing federated learning algorithms for IoT deployments requires evaluating multiple performance factors. In this study, we employ analytical models and simulations to quantify these metrics for TFNN and baseline methods, focusing on their implications for energy consumption, communication overhead, and recognition performance.

### 4.1. Computational Complexity

In federated learning scenarios—particularly within IoT networks constrained by limited computational and power resources—local computational efficiency is critical. The TFNN approach addresses this challenge by eliminating iterative model training, thereby reducing processing demands. Instead, each IoT node performs lightweight local inference using a *k*-NN algorithm on a fixed-size pattern dataset.

The local computational cost at each node in TFNN is primarily determined by the *k*-NN classification step. For a local prototype set $P_{i}$ of size $|P_{i}|$ and input feature vectors of dimension *d*, each node locally computes the distance between the input event pattern and stored patterns, resulting in a per-pattern complexity of

(6) $$O(|P_{i}|\cdot d)$$

Compared to machine learning algorithms that require forward passes through large neural networks, this cost is both low and predictable, making TFNN well suited for microcontrollers and IoT-grade processors. Furthermore, TFNN avoids backpropagation, weight storage, and floating-point intensive operations common in many federated learning algorithms. This significantly reduces the memory footprint and energy consumption at each node—an essential advantage for battery-powered and intermittently connected devices. Overall, TFNN provides a favorable trade-off by enabling decentralized intelligence with minimal local computation, making it highly practical for resource-constrained federated edge environments.

### 4.2. Accuracy Analysis

The classification accuracy of the TFNN approach depends on several interdependent factors, including the number of participating nodes (*m*) and the number of nearest neighbors (*k*). At the local level, each node executes a *k*-NN classifier, with accuracy determined by the quality of its local dataset and the choice of *k*. While smaller values of *k* may lead to overfitting or increased sensitivity to noise, larger values generally improve stability but may reduce specificity.

The size and quality of local data $\left|D_{i}\right|$ directly affect prediction accuracy, with larger local datasets generally improving *k*-NN performance due to better neighborhood coverage. When using *k*-nearest samples for classification, the risk of underfitting or overfitting becomes more pronounced. In extreme cases, *k*-NN may yield unreliable results if $k\geq \left|D_{i}\right|$ or become overly sensitive if k=1. Formula (7) describes the accuracy trend of *k*-NN accordingly:

(7) $${\mathrm{Acc}}_{\mathrm{kNN}}\propto 1-O\left(\frac{1}{\sqrt{n_{i}}}\right)\;(\mathrm{for}\;\mathrm{large}\;n_{i})$$

Consequently, a small *k* results in low bias and high variance, making the classifier sensitive to noise and prone to misclassifications due to outliers or label noise. In contrast, a large *k* provides better generalization with high bias and low variance; however, it may overlook minority classes. In general, an effective choice is *k* ≈$\sqrt{n_{i}}$, where $n_{i}=\left|D_{i}\right|$ represents the number of samples at node *i*.

Moreover, let the local accuracy of the *k*-NN classifier at node *i* be denoted by $h_{k\text{-}\mathrm{NN}}^{\left(i\right)}\left(x\right)$. Then, the local accuracy of the *k*-NN classifier at node *i* is given by

(8) $${\mathrm{Acc}}^{\left(i\right)}=E_{\left(x,y\right)∼D_{i}}\left[I\;\left(h_{k\text{-}\mathrm{NN}}^{\left(i\right)}\left(x\right)=y\right)\right]$$

Under regularity conditions—such as independent and identically distributed samples and the Lipschitz continuity of the conditional distribution—as $n_{i}\to \infty$, the local accuracy converges to

(9) $$\lim_{n_{i}\to \infty }{\mathrm{Acc}}^{\left(i\right)}\geq {\mathrm{Acc}}_{\mathrm{Bayes}}-C\cdot k^{-1/d}$$

where ${\mathrm{Acc}}_{\mathrm{Bayes}}$ denotes the Bayes-optimal accuracy for the classification problem, C>0 is a constant that depends on the underlying data distribution, *k* is the number of nearest neighbors, and *d* is the dimensionality of the feature space.

For practical finite datasets, a tight upper bound described in [44] is given by

(10) $${\mathrm{Acc}}^{\left(i\right)}⪆\left(1-\sqrt{\frac{c}{k}}\right)\cdot {\mathrm{Acc}}_{\mathrm{Bayes}}$$

where ${\mathrm{Acc}}^{\left(i\right)}$ denotes the expected classification accuracy at node *i*, *k* is the number of nearest neighbors, c>0 is a constant that depends on the underlying data distribution and class overlap, and ${\mathrm{Acc}}_{\mathrm{Bayes}}$ is the accuracy of the Bayes-optimal classifier.

In the TFNN approach, the final classification is obtained by aggregating local predictions from *m* IoT nodes using unweighted majority voting, as described in Formula (5). Each node $n_{i}\in N$ independently predicts a class label ${\hat{y}}^{i}$ using a local *k*-NN classifier, and the server or base stations then computes the global output using the same majority voting rule. The resulting global accuracy depends on the statistical properties of the ensemble decision-making process.

Assuming that each node makes an independent prediction with a probability *p* of being correct, the probability that the majority of nodes produce the correct label is given by the binomial cumulative distribution function (CDF):

(11) Accglobal=∑i=⌈m/2⌉mmipi(1−p)m−i

where mi denotes the binomial coefficient, $p^{i}$ represent the probability that exactly *i* nodes predict correctly, and ${\left(1-p\right)}^{m-i}$ represents the probability that the remaining m−i nodes predict incorrectly.

This reflects the classic ensemble effect: as long as individual nodes perform better than random guessing (p>0.5), increasing the number of participating IoT nodes improves the global accuracy, enabling the ensemble to outperform any individual node. In the asymptotic case, with IID data and a sufficiently large number of nodes, the performance of TFNN can approach that of a globally trained *k*-NN model.

### 4.3. Communication Overhead

Minimizing communication overhead is a critical concern for IoT devices operating on limited battery reserves. Generally, federated learning strategies require multiple exchanges of large iterative model parameters between distributed clients and a coordinating server. TFNN, by contrast, streamlines this process through a single-shot classification approach. Each participating node independently executes a lightweight *k*-NN classifier on its locally stored data and transmits only the resulting class labels to the central aggregator at the server or base station.

Let *m* denote the number of participating nodes and *C* the number of classes. Assuming each prediction is encoded as a one-hot vector of length *C* or as an integer requiring $\left\lceil \log_{2}C\right\rceil$ bits, TFNN incurs negligible downlink cost since the datasets are fixed and locally stored. Unlike conventional federated learning, the server does not broadcast global model updates back to the nodes.

In addition to abstract communication complexity, practical physical costs such as radio transmission energy, propagation distance, and receiver wake-up durations must also be considered. Each IoT node transmits a scalar index ${\hat{y}}^{i}\in 0,1,\ldots ,C-1$, requiring $\log_{2}C$ bits, or a one-hot vector of length *C*, requiring *C* bits. Thus, the total uplink communication costs for *m* nodes is given by

(12) $$O\;\left(m\cdot \log_{2}C\right)\;\mathrm{bits}$$

If $d_{i}$ denotes the distance from node *i* to the server or base station and α is the path loss exponent, the per-node transmission energy scales as

(13) $$E_{\mathrm{comm}}^{\mathrm{tx},i}=E_{\mathrm{tx}}\cdot d_{i}^{\alpha }\cdot \log_{2}C$$

Aggregating over all nodes yields the total uplink transmission energy:

(14) $$E_{\mathrm{comm}}^{\mathrm{tx},\mathrm{total}}=\sum_{i=1}^{m}E_{\mathrm{tx}}\cdot d_{i}^{\alpha }\cdot \log_{2}C$$

In duty-cycled networks, transceivers consume a wake-up energy cost ($E_{\mathrm{wake}}$) in addition to transmission or reception energy. If $E_{\mathrm{rx}}$ denotes the per-bit reception energy, the server’s total reception energy is given by

(15) $$E_{\mathrm{comm}}^{\mathrm{rx}}=m\cdot E_{\mathrm{rx}}\cdot \log_{2}C$$

Including wake-up costs, the per-classification communication energy is expressed as

(16) $$E_{\mathrm{total}}^{\mathrm{node},i}=E_{\mathrm{wake}}^{\mathrm{tx}}+E_{\mathrm{tx}}\cdot d_{i}^{\alpha }\cdot \log_{2}C$$

(17) $$E_{\mathrm{total}}^{\mathrm{BS}}=m\cdot \left(E_{\mathrm{wake}}^{\mathrm{rx}}+E_{\mathrm{rx}}\cdot \log_{2}C\right)$$

By communicating only a single scalar class label per IoT node in one communication round, TFNN achieves a substantially lower communication overhead than standard federated learning, making it well suited for low-power, bandwidth-constrained IoT deployments.

### 4.4. Privacy Considerations

Similar to conventional federated learning methods that exchange model updates, TFNN transmits only a scalar class label from each node for each inference. This substantially reduces the information available to potential eavesdroppers and mitigates risks associated with model inversion or data reconstruction attacks. Raw sensor data or detailed model parameters are never shared across the network. However, in scenarios where the class label space itself may reveal sensitive information (e.g., rare disease diagnosis), even label-only transmission could expose limited contextual details to a malicious observer. In such cases, additional privacy-enhancing techniques—such as label perturbation or differential privacy—can be integrated without significantly affecting TFNN efficiency.

## 5. Results

The on-board battery energy of IoT devices is predominantly consumed during communication. Therefore, this study focuses on evaluating the communication efficiency and classification accuracy of the TFNN approach by comparing its performance with traditional federated learning and pattern recognition methods, specifically AdaptiveFL, FedProx, SCAFFOLD, FedAvg, and the *k*-NN algorithm, respectively. The evaluation of TFNN was conducted using a custom simulator developed in MATLAB 2024b and Python 3.13.3 [56,57]. The experiments employed three widely used benchmark datasets—MNIST [58], CIFAR-10, and CIFAR-100 [59]—across various experimental setups. The CIFAR-10 dataset contains 60,000 color images of size 32×32 pixels across 10 classes, while the CIFAR-100 dataset comprises 100 classes with 600 images per class. MNIST consists of handwritten digits (0–9, 10 classes), where each image comprises 28×28 grayscale patterns.

**Figure 1:**
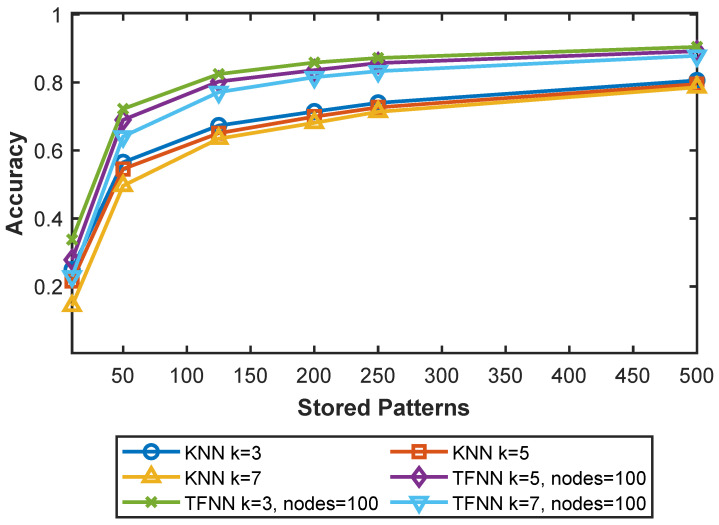
Accuracy comparison between traditional *k*-NN and TFNN using 100 IoT nodes, evaluated at k=3, k=5, and k=7 while increasing the number of stored patterns from 10 to 500 patterns.

Figure 1 illustrates the classification accuracy of the *k*-NN algorithm with different values of *k* compared with the proposed TFNN approach, using 100 IoT nodes, and varying the number of IID stored training patterns from 10 to 500 from the MNIST dataset. As the number of stored patterns increases, the accuracy of both methods improves; however, TFNN consistently achieves higher accuracy than *k*-NN across all configurations. While *k*-NN serves as a simple and interpretable baseline, TFNN leverages lightweight collaborative inference in a federated setting, resulting in substantially improved performance. Notably, TFNN achieves approximately 90% accuracy when the number of stored patterns reaches 500.

**Figure 2:**
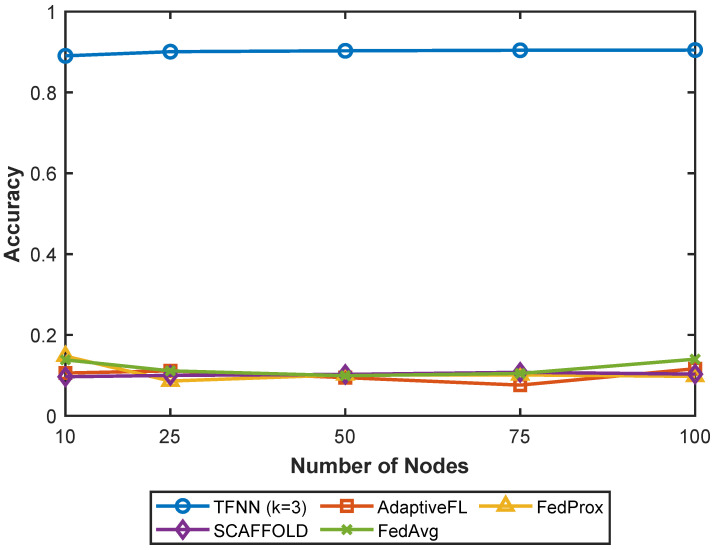
Single-shot (one communication round) classification accuracy comparing TFNN (k=3) with AdaptiveFL, FedProx, SCAFFOLD, and FedAvg.

Figure 2 compares the single-shot classification accuracy of the proposed TFNN (k=3) approach with AdaptiveFL, FedProx, SCAFFOLD, and FedAvg in IoT networks ranging from 10 to 100 nodes, with each node storing 500 IID MNIST patterns. The results show that TFNN consistently outperforms all federated learning baselines, achieving approximately 90% accuracy across all network sizes and thereby demonstrating strong communication efficiency—an essential feature for resource-constrained IoT environments.

**Figure 3:**
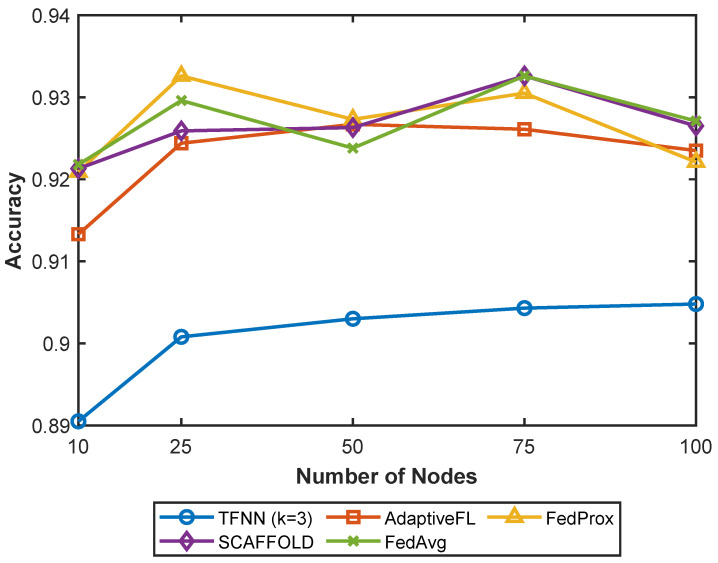
Single-shot classification accuracy of TFNN (k=3) compared with AdaptiveFL, FedProx, SCAFFOLD, and FedAvg after 200 communication rounds.

Figure 3 presents the accuracy of the federated baselines after 200 communication rounds compared with the single-shot TFNN result. While the baselines achieve approximately 92%, this requires nearly 200 additional client–server exchanges beyond TFNN’s one-round operation and yields only about a 2% improvement in accuracy. These results underscore TFNN’s ability to achieve competitive accuracy with orders-of-magnitude lower communication overhead—and, by implication, lower communication energy—in practical IoT deployments.

Table 2, Table 3 and Table 4 present the classification accuracy of AdaptiveFL, FedProx, SCAFFOLD, FedAvg, and the proposed TFNN (k=3) under both IID and Non-IID partitions of the MNIST, CIFAR-10, and CIFAR-100 datasets. The simulations were conducted over 200 communication rounds with network sizes ranging from 10 to 100 IoT nodes, where each node stored 500 patterns.

Whilst using the MNIST dataset, all baseline algorithms achieved accuracies in the range of 92–93% under IID conditions, confirming strong convergence on relatively simple data. TFNN attained accuracy of approximately 80–90%, representing a modest trade-off in accuracy in exchange for an approximately 200-fold reduction in communication energy, as TFNN operates in a single-shot manner.

On the CIFAR-10 dataset, baseline federated learning methods achieved accuracies clustered around 36–39% under IID conditions, whereas TFNN remained consistently lower (≈23–25%). Under non-IID partitions, accuracy remained low for both the baseline federated learning algorithm and TFNN. The CIFAR-100 dataset is, in general, challenging, with all algorithms—including TFNN—achieving only about 10% accuracy or less. The reduced performance of TFNN on CIFAR-10 and CIFAR-100 can be attributed to the higher feature dimensionality and greater class complexity of these datasets.

Overall, the results reveal a clear energy-efficiency and accuracy trade-off. TFNN achieves competitive accuracy while requiring only a single communication round, thereby drastically reducing communication costs and, consequently, energy consumption compared with classical federated learning algorithms. These findings position TFNN as a viable lightweight alternative for resource-constrained IoT and WSN deployments, where communication efficiency is critical and absolute accuracy may be moderately sacrificed. However, the utilization of TFNN needs to take into account the complexity of the event patterns in order to maintain acceptable accuracy.

## 6. Conclusions

This study proposed TFNN, a lightweight and communication-efficient approach to federated learning tailored for resource-constrained IoT and WSN environments. Unlike conventional federated learning algorithms that require tens to hundreds of communication rounds and the exchange of large model parameter updates, TFNN performs single-shot pattern recognition by transmitting only local class labels from IoT nodes to the server or base station. Consequently, TFNN significantly reduces the energy consumed in communication compared with traditional federated learning while enabling federated pattern recognition in a single communication round.

The simulation results demonstrated that TFNN achieves competitive classification accuracy while reducing communication cost by up to two folds compared with baseline federated learning methods. This makes TFNN particularly promising for battery-powered and bandwidth-constrained IoT deployments, where extending network lifetime is more critical than achieving absolute accuracy.

Future studies will focus on refining and optimizing TFNN, specifically addressing its sensitivity to complex datasets by integrating lightweight feature extraction or prototype-sharing mechanisms. This is expected to enhance TFNN’s ability to capture discriminative representations in high-dimensional data. Furthermore, deploying TFNN in practical IoT environments will allow for an exploration of its full potential across diverse real-world scenarios. Such extensions will further establish TFNN as a scalable and energy-efficient solution for next-generation IoT systems.

## Figures and Tables

**Table 1:** Comparison of federated learning algorithms FedAvg, FedProx, SCAFFOLD, and AdaptiveFL.

Feature	FedAvg	FedProx	SCAFFOLD	AdaptiveFL
Proposed by	McMahan et al., 2017 [8]	Li et al., 2020 [53]	Karimireddy et al., 2020 [47]	Jia et al., 2024 [54]
Convergence rate	Moderate (suitable for IID)	More stable than FedAvg on non-IID; moderate	Faster on non-IID (drift corrected)	Faster than FedAvg under heterogeneous settings
Computation cost (per round)	Low	Low–medium (proximal term)	medium (control updates)	Medium (slice/merge + subnet training)
Communication rounds	High	High–moderate	Fewer than FedAvg on non-IID	Moderate; fewer than FedAvg when device heterogeneity is well matched
Robustness to system/data heterogeneity	No	Partial (regularizes objective)	Yes (corrects drift)	Yes (adapts model to device)
Accuracy	High on IID; drops on non-IID	More stable than FedAvg on non-IID	High; provable convergence on non-IID	Competitive; benefits from adaptive modeling

**Table 2:** Accuracy after 200 communication rounds using MNIST dataset under IID vs. non-IID partitions across varying numbers of participating nodes.

Nodes	AdaptiveFL		FedProx		SCAFFOLD		FedAvg		TFNN (k = 3)	
	IID	Non-IID	IID	Non-IID	IID	Non-IID	IID	Non-IID	IID	Non-IID
10	91.33%	31.63%	92.09%	51.30%	92.13%	52.14%	92.18%	48.91%	89.05%	32.93%
25	92.44%	37.55%	93.26%	80.23%	92.59%	78.00%	92.96%	80.45%	90.08%	42.30%
50	92.63%	52.55%	92.38%	80.60%	92.44%	77.39%	93.26%	75.98%	90.30%	39.41%
75	92.61%	86.66%	93.05%	88.73%	92.67%	88.84%	93.26%	88.71%	90.43%	47.72%
100	92.35%	86.00%	92.21%	88.61%	92.65%	89.94%	92.71%	88.98%	90.48%	73.84%

**Table 3:** Accuracy after 200 communication rounds using CIFAR-10 dataset under IID vs. non-IID partitions across varying numbers of participating nodes.

Nodes	AdaptiveFL		FedProx		SCAFFOLD		FedAvg		TFNN (k = 3)	
	IID	Non-IID	IID	Non-IID	IID	Non-IID	IID	Non-IID	IID	Non-IID
10	35.73%	13.80%	36.83%	19.68%	36.64%	10.00%	36.45%	17.93%	23.62%	18.87%
25	35.41%	13.53%	37.06%	14.76%	38.23%	9.94%	39.20%	14.91%	24.28%	18.59%
50	35.78%	12.98%	38.60%	17.58%	36.81%	10.00%	36.45%	15.39%	24.75%	23.19%
75	36.67%	25.53%	37.09%	31.44%	36.57%	19.74%	37.81%	32.44%	25.37%	27.61%
100	36.46%	24.29%	36.85%	31.64%	38.60%	21.34%	36.82%	32.70%	25.23%	27.61%

**Table 4:** Accuracy after 200 communication rounds using CIFAR-100 dataset under IID vs. non-IID partitions across varying numbers of participating nodes.

Nodes	AdaptiveFL		FedProx		SCAFFOLD		FedAvg		TFNN (k = 3)	
	IID	Non-IID	IID	Non-IID	IID	Non-IID	IID	Non-IID	IID	Non-IID
10	4.95%	1.29%	7.09%	2.34%	8.34%	1.00%	7.26%	2.47%	7.00%	1.69%
25	4.84%	1.69%	6.51%	3.14%	6.68%	1.22%	8.73%	3.13%	8.87%	1.60%
50	6.69%	3.36%	7.57%	4.23%	8.17%	0.99%	7.87%	3.84%	9.70%	5.36%
75	6.54%	4.15%	8.80%	5.52%	8.23%	0.97%	5.07%	4.63%	10.37%	6.90%
100	6.11%	3.30%	7.26%	5.32%	6.35%	1.00%	6.27%	4.57%	10.15%	7.28%

## Data Availability

Data are contained within the article.
